# Establishing the added benefit of measuring MMP9 in FOB positive patients as a part of the Wolverhampton colorectal cancer screening programme

**DOI:** 10.1186/1471-2407-9-36

**Published:** 2009-01-28

**Authors:** Sue Wilson, Taina Taskila, Tariq Ismail, Deborah D Stocken, Ashley Martin, Val Redman, Michael Wakelam, Ian Perry, Richard Hobbs

**Affiliations:** 1 Primary Care Clinical Sciences, The University of Birmingham, Edgbaston, Birmingham, B15 2TT, UK; 2 University Hospital Birmingham Foundation NHS Trust, Queen Elizabeth Hospital, Birmingham, B15 2TH, UK; 3 Cancer Research UK Institute for Cancer Studies, The University of Birmingham, Edgbaston, Birmingham, B15 2TT, UK; 4 Wolverhampton Bowel Cancer Screening Programme, Beynon Centre, New Cross Hospital, Wednesfield Road, Wolverhampton, WV10 0QP, UK

## Abstract

**Background:**

Bowel cancer is common and a major cause of death. The NHS is currently rolling out a national bowel cancer screening programme that aims to cover the entire population by 2010. The programme will be based on the Faecal Occult Blood test (FOBt) that reduces mortality from colon cancer by 16%. However, FOB testing has a relatively low positive predictive value, with associated unnecessary cost, risk and anxiety from subsequent investigation, and is unacceptable to a proportion of the target population. Increased levels of an enzyme called matrix metalloproteinase 9 (MMP9) have been found to be associated with colorectal cancer, and this can be measured from a blood sample. MMP9 has potential for detecting those at risk of having colorectal cancer. The aim of this study is to assess whether MMP9 estimation enhances the predictive value of a positive FOBt.

**Methods and design:**

FOBt positive people aged 60–69 years attending the Wolverhampton NHS Bowel Cancer Screening Unit and providing consent for colonoscopy will be recruited. Participants will provide a blood sample prior to colonoscopy and permission for collection of the clinical outcome from screening unit records. Multivariate logistic regression analyses will determine the independent factors (patient and disease related, MMP9) associated with the prediction of neoplasia.

**Discussion:**

Colorectal cancer is a major cause of morbidity and mortality. Pilot studies have confirmed the feasibility of the national cancer screening programme that is based on FOBt. However, the test has high false positive rates. MMP9 has significant potential as a marker for both adenomas and cancers. This study is to examine whether using MMP9 as an adjunct to FOBt improves the accuracy of screening and reduces the number of false positive tests that cause anxiety and require invasive and potentially harmful investigation.

## Background

About one in 20 people in the UK will develop colorectal cancer during their lifetime [1]. It is the third most common cancer in the UK, and the second leading cause of cancer death, with over 15,000 people dying from colorectal cancer each year [2]. Colorectal cancer incurs an annual expenditure of more than £300 million in surgical, adjuvant, and palliative treatment [3]. As the population ages these costs are set to increase. Reduced costs of treatment could be achieved by earlier diagnosis. However, most cases are diagnosed at a late stage which is strongly associated with poorer survival; the five year overall survival rate of colorectal cancer is only 48% [4]. Benefits, in terms of improved survival, improved quality of life and reduced treatment costs, could be accrued by earlier diagnosis.

FOBt screening can detect colorectal cancer at an early stage when treatment is more likely to be effective. It also provides an opportunity to identify precursors to invasive disease, polyps, which can be removed during colonoscopy and reduce the risk of colorectal cancer developing. Randomised controlled trials suggest that colorectal screening has the potential to reduce colorectal cancer mortality by 16% [5]. In the light of the evidence from these trials the NHS has introduced a national bowel cancer screening programme [6]. The first UK bowel cancer screening site started screening men and women aged 60–69 years in July 2006 and national coverage is expected to be achieved by 2010. The programme uses the Faecal Occult Blood test (FOBt). Participants in screening, who have a positive FOBt, are then invited for colonoscopy. Pilot evaluations, in Scotland and Rugby, confirmed the feasibility of the national screening programme; however, they also demonstrated relatively low acceptability of FOBt with uptake rates of only 58.5%[7] and 52% [8] in the first and second rounds of screening respectively.

Recent results from the NHS Bowel Cancer screening pilot studies demonstrate that, FOBt testing has a sensitivity of 57.7%, with a positive predictive value of 5.3% for cancer and 38.8% for neoplasia [8]. The low positive predictive value means that although all FOBt positive results require investigation via colonoscopy, many of these are false positive results with the associated cost, risk and anxiety. Colonoscopy carries a risk of bowel perforation of 1 in 1,500 [9].

Therefore, although FOBt screening is likely to reduce the mortality attributable to bowel cancer there is an urgent need to improve the screening test, ideally to increase the positive predictive value. Serum matrix metalloproteinase 9 (MMP9) are proteolytic enzymes that are associated with tissue remodelling in normal and pathological processes [10]. Over-expression of MMP9s has been correlated with progression in many tumour types, including colorectal cancer [11-13]. Our pilot suggests that MMP9 has potential in detecting those at risk of having colorectal cancer as it demonstrates a high specificity and positive predictive value [11].

### Pilot work

A pilot study of 300 patients attending the Queen Elizabeth Hospital colorectal clinic was performed. Twenty seven significant adenomas and 63 malignancies were identified in the study population. Patients had a standard assessment, by proforma-led history and examination, with rigid sigmoidoscopy to the recto-sigmoid junction. The patients gave serum samples for analysis, and referral for specialist investigations occurred as per the clinic-protocol. Forty-six "normal" volunteers also donated serum. ELISAs were done on each serum sample, and the results were compared with the clinical diagnosis. The median sMMP9 concentration was 443 ngml^-1^. The model accurately predicted neoplasia in 77.3% of cases (sensitivity 77.9%, specificity 77.1%, positive predictive value (PPV) 44.6%, and negative predictive value (NPV) 95.8%) in a population with 30% prevalence of disease [11].

The results of this pilot suggest that MMP9 may be an effective secondary screening test, but this study was completed in a selected population. The performance of a diagnostic test can vary in populations with different severity and prevalence of disease and, therefore, the acceptability and accuracy of MMP9 needs to be established in the target population [14].

### Study aim

The aim of the study is to assess whether the addition of MMP9 testing enhances the predictive value of a positive FOBt.

## Methods

Study design: evaluation of a diagnostic test.

Setting: Wolverhampton NHS Bowel Screening Unit

Subjects: Participants in the NHS Bowel Cancer Screening Programme who are FOBt positive and attending for colonoscopy.

Those consenting to colonoscopy will be given a patient information leaflet about the study. Prior to colonoscopy, all those expressing an interest, will be seen by a member of the research team who will answer any outstanding questions and seek consent.

Intervention: Participation will entail the provision of a blood sample prior to colonoscopy, completion of a baseline questionnaire (demographics, symptoms, and duration of symptoms) and permission for data collection (final diagnosis) from screening unit records.

A trained nurse/phlebotomist will take two blood samples (10 ml) to enable MMP9 determination and for a routine haematology profile: Haemoglobin (Hb), Whole blood cell count (WBC) and Red cell count (RCC). Blood samples will be kept on ice and spun within 4 hours of collection. Local storage will be at -80C until transported to the Department of Cancer Studies, University of Birmingham, for analysis.

Outcomes assessment: diagnosis collected from screening office records. A technician, blinded to the symptoms, signs, or diagnosis of the patient, will determine MMP9 levels by ELISA. Duplicate determinations will be performed upon coded samples.

### Procedures for handling data

All electronic data will be processed using password protected systems and paper data will be stored securely in locked cabinets/rooms. Participant identification numbers will be employed to enable separate storage/processing of name and address data. Identifiable data will be handled only by University of Birmingham staff working on this study who are trained in policies and procedures related to confidentiality.

### Sample size estimation

In a 12 month period, 200 people are expected to have a positive FOBt as part of the screening programme and be referred to Wolverhampton Screening Centre.

Our pilot data indicates that elevated MMP9 has a high sensitivity and negative predictive value. The potential of measuring MMP9 in FOBt positive people will be to reduce the number of unnecessary colonoscopies i.e. a high NPV is required. The pilot data indicated that 126 test negative patients are needed to detect a NPV of 95% with 5% precision and 2-sided alpha of 1%. Based on this data, a total of 209 patients are required to enable 91% and of 31% prevalence.

### Analyses

Patients will be classified into high risk (invasive adenocarcinoma or high risk polyps) and lower risk diagnostic groups. The association between possible risk factors and diagnosis will be determined based on odds ratios (OR). Multivariate logistic regression analyses will determine the independent factors for prediction of high risk disease excluding MMP9 and provide adjusted OR accordingly. The influence of MMP9 will then be assessed by the addition of MMP9 to the derived logistic regression model.

Accuracy and comparison of the predictive models, including and excluding MMP9, will be assessed by the estimates of sensitivity, specificity, proportion of false positives and negatives and overall percentage of correct predictions. Receiver operating curves (ROC) and area under the curve (AUC) of the ROC will be presented for each of the models, including and excluding MMP9, as an indication and comparison of diagnostic ability.

### Bias and confounding factors

There will be duplicate determination of the serum MMP9 level and dual data entry. Information on potential confounders, for example injuries or chronic illnesses that may lead to a raised serum MMP9, will be collected by the research nurse. The analyses will be adjusted to take account of any confounders or selection bias. All blood samples will be analysed in the same laboratory to ensure standardisation of measurement and reporting. The technician doing the MMP9 ELISA assay will be blinded to the symptoms, signs or diagnosis of the patient.

### Ethical approval

This study has been approved by the Black Country Research Ethics Committee, 6^th ^August 2007. REC reference number: 07/H1202/72.

## Discussion

Colorectal cancer is a major cause of morbidity and mortality. Most colorectal cancers arise from adenomas and could be detected early by screening. The national bowel cancer screening programme uses FOBt. Even though FOB reduces mortality the test has high false positive rates. Increased levels of an enzyme called matrix metalloproteinase 9 (MMP9) has significant potential as a marker for both adenomas and cancers, and this can be measured from a blood sample. This study is to examine whether using MMP9 as an adjunct to FOBt can improve the accuracy of screening and reduce the number of false positive tests that cause anxiety and require invasive and potentially harmful investigation.

## Competing interests

The authors declare that they have no competing interests.

## Authors' contributions

SW, TI, MW, RH and IP designed the study. TT drafted the manuscript. MW is responsible for MMP9 estimation and IP supervised the colonoscopies; TI has responsibility for clinical and DDS is the statistician for the study. VR is responsible for data collection and TT has responsibility for data validation. All authors read and approved the final manuscript.

## Pre-publication history

The pre-publication history for this paper can be accessed here:

http://www.biomedcentral.com/1471-2407/9/36/prepub
